# Do programme coordinators contribute to the professional development of residents? an exploratory study

**DOI:** 10.1186/s12909-022-03447-y

**Published:** 2022-05-18

**Authors:** Mayumi Aono, Haruo Obara, Chihiro Kawakami, Rintaro Imafuku, Takuya Saiki, Michael A. Barone, Yasuyuki Suzuki

**Affiliations:** 1 Division of Medical Education, Graduate School of Medicine, Gifu University, 1-1 Yanagido, Gifu, 501-1194 Japan; 2 St Luke’s International University, 10-1 Akashi-cho, Chuo-ku, Tokyo, 104-0044 Japan; 3 Department of Medicine, Okinawa Chubu Hospital, 281 Miyazato, Uruma, 904-2243 Japan; 4 Department of Pediatrics (Adjunct), Johns Hopkins University School of Medicine, 733N Broadway, Baltimore, MD 21205 USA

**Keywords:** Programme coordinator, Educational role, Resident, Professional identity formation

## Abstract

**Background:**

With the development of training programmes for health professions, the role of programme coordinators has become increasingly important. However, their role in providing educational support for the professional development of resident trainees has not been investigated well. This study aimed to qualitatively analyse the involvement of programme coordinators in educational support for residents.

**Methods:**

Semi-structured reflective writing on ‘support for residents’ was collected from programme coordinators in teaching hospitals in Japan in 2017–18 using a web-based questionnaire. Descriptions were qualitatively analysed thematically, using the professional identity formation (PIF) framework.

**Results:**

A total of 39 cases of “support for residents” by 31 coordinators were analysed. We found that residents most commonly faced prior personal problems, including mental health issues and insufficient social skills/unprofessional behaviour. A thematic analysis revealed that coordinators played a variety of educational roles: 1) requesting supervisors to reconsider their teaching; 2) protecting residents from the negative influence of clinical experiences; 3) facilitating residents’ self-assessment and confidence; 4) creating a safer learning environment; 5) providing support for prior personal problems through 5–1) fostering a better atmosphere for the mental health of residents, and 5–2) intervening for residents with insufficient social skills/unprofessional behaviour; 6) providing support for isolated residents; and 7) preventing problems with peers.

**Conclusions:**

This study identified seven educational roles of programme coordinators for residents from a standpoint of PIF of residents. Based on these findings, four valuable attributes for coordinators were established: non-hierarchical relationships with residents, parenting attitudes, sensitivity to residents’ changes, and the perspective of the citizen and a member of the public. These attributes would underpin coordinators’ educational roles and facilitate the professional development of residents. This study provides a basis for defining and revising the role profiles of programme coordinators, and for improving staff development.

**Supplementary Information:**

The online version contains supplementary material available at 10.1186/s12909-022-03447-y.

## Background

For improved healthcare, training programs for medical professionals have been developed worldwide. Accordingly, supervising doctors and healthcare professionals need to have skill sets to support for trainees through various staff development programmes [1]. Among these healthcare professionals, non-medical program coordinators who provide administrative support to and manage training programmes have recently been considered as integral members of the leadership team of residency programmes, and many programme directors in the US rely on them [2]. Owing to rapid changes in knowledge and skills required for residency programmes, those who can be a management change agent and maximize the effectiveness in a working relationship are now necessary for the evolving role of program coordinators [3]. Defining the roles and functions of programme coordinators is becoming increasingly important, and O’Sullivan et al. reported that an educational development program including counselling and institutional assistance of trainees was effective for both program directors and coordinators [4]. Dubois et al. stated in their perspective article that the title of “programme coordinator” no longer seems adequate to represent the position [5]. Instead, they proposed that *programme manager*, *administrator*, *educational specialist* [6], *or medical education manager* [7] seemed appropriate. They also pointed out that a higher level of coordinator competency would be necessary to improve the quality of the training programmes and provide support for residents experiencing training difficulties [5]. A formal certification program for coordinators/administrators launched in the USA for the purpose of promoting excellence in management of graduate medical education programmes [8].

As programme coordinators have direct contact with residents throughout the training period, coordinators would also have frequent opportunities to care and provide support for residents with difficulties or unprofessional behaviours that may result in an adverse effect on their professional development. According to Holmboe et al., nurses and ancillary staff are stable members of a clinical unit and serve as vital threads of continuity for trainees and faculty members [9]. Previous studies have revealed residents faced difficulties with their supervising doctors with a hierarchical relationship but these residents were supported by team members of other health professions with more non-hierarchical attitudes [10]. However, these opportunities and the specifics of the educational support provided by programme coordinators for the professional development of trainees have not been investigated and are not typically reflected on the job description. Job descriptions of programme coordinators have been reported from various fields, including surgery [2], radiology [11] and orthopaedics [12], where their roles have expanded beyond administrative functions, but the educational and supportive roles played by coordinators have not been well described.

Some previous studies suggest expanding coordinators’ roles, especially on the educational and supportive front. In a study of 22 female coordinators managing undergraduate clinical training, Hu et al. [13] revealed that coordinators’ roles related to emotional and informal support, and related to their personal orientation. Some were like nurturing parents and identified with the ‘mother hen’ persona, referring to the students as ‘chicks’. They argue that this image has come from societal expectations of women as caring beings responsible for the welfare and positive outcomes for the young [13], indicating the educational and supportive roles of coordinators, while the outcomes for the young relate to their professional development. Nickels et al. provided another potential educational role for program coordinators, using a questionnaire survey for non-physician staff members including administrative staff, and clarified that many of them felt comfortable providing feedback on the professional behaviour of residents [14]. We have earlier reported that coordinators in Japan recognized the importance of educational support roles, including parental roles, for trainees [15].

Professional identity formation (PIF) of medical trainees has been recognized as an important issue in medical education. To date, many scholars have examined and conceptualised the processes of PIF in medical education. For example, Cruess et al. propose that ‘a principal goal of medical education is the development of a professional identity’, through complex socialization process, and learners gradually ‘think, act, and feel like a physician’ as they are establishing a professional identity [16]. Cruess et al. also provided the schematic representation of PIF and a number of socialization factors influencing identity formation, including role models/supervising doctors, clinical/non-clinical experiences, clinical peers and health professionals, patients, families and friends of trainees, and the public [17]. These factors dependently and independently affect the trainees’ PIF process. Bhat et al. identified nine threshold concepts that trainees may be struggling with or should overcome; such as the burden of responsibility, the plurality of roles, and uncertainty of medicine; many of these thresholds are deeply related to PIF [18]. Snell reviewed various methods and individuals supporting PIF at the postgraduate level [19]. Programme coordinators who support trainees throughout the training period would be additional professionals who play an important but underestimated role during the trainees’ PIF process. Thus, the role of programme coordinators as a supporter and educator of trainees who are undergoing PIF is worthy for further investigation.

This study aimed to identify the possible educational roles of programme coordinators through a qualitative analysis of their involvement in educational supports for residents from their perspectives, using a PIF model as a theoretical framework. Specifically, the following research questions were formulated:

*How are programme coordinators involved in the professional development of trainees?; and how do they support the professional development of trainees?*

### Theoretical framework

The socialization factors of PIF proposed by Cruess et al. [17, 20] were used as the theoretical framework. Cruess et al. established a schema of PIF according to which novice students/trainees develop their professional identity through various experiences, which are categorized into socialization factors [17]. In our study, each case described by the coordinators was qualitatively analysed based on the following socialization factors: 1) role models/supervisors/mentors, 2) clinical/non-clinical experiences, 3) formal teaching/self-assessment, 4) learning environment, 5) prior personal problems, 6) isolation from family, friends, and peers, and 7) problems with patients and peers. Among these factors, ‘role models/supervisors/mentors’ and ‘clinical/non-clinical experiences’ are major factors and strongly influence residents’ PIF. ‘Prior personal problems’ is modified from the original factor ‘existing personal identities’ [17] and includes mental health problems, insufficient social skills, and unprofessional behaviours. ‘Isolation from family, friends, and peers’ is necessary during professional development. Relationships with peers, health professionals, and patients also have a significant impact on the promotion or inhibition of PIF.

## Methods

### Setting

This study analyses the interaction of residents with programme coordinators in Japan. Medical training in Japan includes a six-year undergraduate medical school programme [21], followed by a two-year mandatory junior residency programme introduced in 2004 [22, 23]. Residents rotate through internal medicine, surgery, emergency medicine, paediatrics, obstetrics and gynaecology, psychiatry, community medicine, and electives [23]. Junior residency programmes are accredited by the Ministry of Health, Labour, and Welfare of Japan, and each teaching hospital must deploy a certain number of qualified supervising doctors. The qualifications, competencies, and role profiles of programme coordinators in this context are not well-established and their roles may vary in every case, from only administrative work to broader responsibilities such as mental support for residents, support for supervisors, and staff development.

### Study design

We conducted a qualitative thematic analysis [24, 25] of the formal and informal educational support provided by programme coordinators to residents across Japan in 2017–18, employing PIF socialization factors [17, 20] as the theoretical framework. We used a web-based semi-structured questionnaire to collect coordinators’ responses on their experiences in supporting residents (Table 1). The instrument was designed as case-based, wherein coordinators described an example event in which they provided support, including general advice, advice on professional life and career, supporting residents with mental health issues, negotiation with supervisors and hospital administration, improvement of the learning environment, and other support for residents.

**Table 1:** Case description in semi-structured web questionnaire

Please describe impressive cases in which you gave advice/support for residents’ personal/professional development. Not only include cases within your official responsibility but also cases outside your responsibility carried out as a senior or relative of the resident.	
Case	Years after graduation, gender
Problem	What was the resident’s challenge or problem? How did you notice or become aware of the issue?
Support	What advice did you provide to the resident? How did you support him/her?
Results	Describe any change in the behaviour, thoughts, the impression of the resident, and changes in the learning environment. Describe any changes made by coordinators, supervisors, programme directors, or the organization.
Comments	Provide any additional comments you feel are relevant.

### Participants

We sent letters of invitation for this research to the administrative offices of 315 teaching hospitals for junior residency programmes qualified by the Ministry of Health, Labour and Welfare of Japan and Japan Council for Evaluation of Postgraduate Clinical Training (JCEP), across urban and rural, and public and private hospitals in Japan. In an attempt to maximize the number of respondents, an invitation letter from the JCEP president was attached. Sixty individual coordinators who had participated in our previous study were also invited directly [15]. Finally, 43 coordinators (28 female and 15 male) from 38 hospitals (12 university hospitals and 26 general hospitals) agreed to participate in this study; 19% of them had worked as coordinators for more than six years, 29% for three to six years, and 52% for less than three years.

### Data collection

An anonymous, case-based semi-structured questionnaire was used to collect data from the participants via the web (Table 1). We developed it to ensure confidentiality, and considering the potential sensitivity of reported cases, to minimise hesitation in reporting experiences. Of the 43 initial participants, 31 responded to the web-based questionnaire (response rate 72%) and reported 44 resident cases.

### Data analysis

The research team comprised seven researchers from different backgrounds: a coordinator (MA), a general practitioner (HO), a nurse educator (CK), an education researcher (RI) and medical educators (TS, MAB, YS). The researchers reviewed case descriptions multiple times to better interpret what the participants reported (familiarization with data phase [24]). Two researchers (MA and YS) were independently involved in the initial coding, then MA, YS, and MAB cross-checked their interpretation and analysis (coding phase/generating initial themes phase [24]). The themes and findings were reviewed, discussed, and refined by all members of the research team (reviewing themes phase/defining and naming themes phase [24]), and all researchers came to a consensus on all final themes.

## Results

### Coordinator support

Five of the 44 collected cases of resident support by programme coordinators were omitted due to insufficient information, and 39 cases were analysed. The supplementary table shows the categorization of socialization factors, issues of residents, and responses of coordinators. Content analysis revealed that prior personal problems, including mental issues and insufficient social skills/unprofessional behaviours, were the most frequent (16 cases), followed by isolation from family/friends/peers (6 cases), problems with peers (6 cases), role models (supervisors)/mentors (4 cases), formal teaching/self-assessment (4 cases), learning environment (2 cases) and clinical experiences (1 case).

### Thematic analysis of support by coordinators

Key roles of and educational support by coordinators emerged for the seven PIF socialization factors [17] through the thematic analysis (Table 2).

**Table 2:** Educational roles of programme coordinators

Roles of programme coordinators	Socialization factors of PIF
1. Requesting supervisors to reconsider their teaching	Role models/supervisors/mentors
2. Protecting residents from the negative influence of clinical experiences	Clinical/non-clinical experiences
3. Facilitating residents’ self-assessment and confidence	Formal teaching/self-assessment
4. Creating a safer learning environment	Learning environment
5. Providing support for prior personal problems 5–1. Fostering a better atmosphere for the mental health of residents 5–2. Intervening for residents with insufficient social skills/unprofessional behaviour	Prior personal problems
6. Providing support for isolated residents	Isolation from family/friends/peers
7. Preventing problems with peers	Problems with patients and peers

#### 1. Requesting supervisors to reconsider their teaching

Some supervisors were overly strict or had opposing opinions and severe supervision attitudes. The strict attitude and opposing opinions of supervisors confused residents and created an awkward relationship between supervisors and residents, resulting in the inhibition of professional development. The coordinators were sensitive to the residents’ stressors and behaviours, noticing their mood changes and responding quickly to improve their relationships with supervisors. They requested supervisors to reconsider their strict teaching style, provided an opportunity for communication between them and the residents, and suggested a review of the teaching manual.

*She was a tough resident physically and mentally and she seemed to communicate with her strict supervisor well, however, I* (the coordinator) *felt that she was under stress based on her facial expression. I talked to her and the supervisor and arranged the opportunity to for them to talk and reflect with each other. I also conveyed her a message to reassure her that I will take care of her and she can say anything to me at any time. (Case 3).*

#### 2. Protecting residents from the negative influence of clinical experiences

Powerful clinical experiences, such as medical errors and patient deaths, had a strong influence, both positive and negative, on the professional development of residents. The coordinators carefully watched, listened to, and instructed residents who had a particularly negative clinical experience to protect and support them. They also facilitated supervisors’ reflection and review of daily teaching.

*The resident had a medical accident that resulted in the death of a patient, and he took leave from work for a while. A hospital team including me* (the coordinator) *was organized for his care. I made a lot of efforts, including listening to him and watching him. The relationship between the resident and the supervisor was reviewed, and the supervisor said that he did not care for the resident enough and left him alone in daily work. The team members worked together to provide psychological support and protect the resident. He completed the training as planned, and he is now very active. (Case 5).*

#### 3. Facilitating residents’ self-assessment and confidence

Residents sometimes had low confidence in their clinical competence and future career. The coordinators noticed subtle changes in their behaviour and appearance and speculated decreased self-confidence. Coordinators reassured the residents, made arrangements for skill training to facilitate and improve their confidence, and created an amiable atmosphere.

*The resident was not confident with her blood sampling technique, and she looked a little depressed. I* (the coordinator) *told her, when she came into my office, that no resident can do blood sampling perfectly from the beginning. I lent her a practice kit for blood sampling and secured a space for her to practice freely. She seemed to become confident, and she came and asked me to lend other practice kits without hesitation thereafter. (Case 7).*

#### 4. Creating a safer learning environment

High workload and stress, and hostile and exclusionary human relationships in the learning environment adversely impact the professional development of residents. The coordinators proposed improvements for resident workload to medical directors and acted as a bridge between the residents and medical team members. They also proposed a review of the resident-centred instructional guide to improve the learning environment.

*The resident seemed overworked, and had trouble with his colleagues. I* (the coordinator) *noticed his dissatisfaction from his tone of voice and facial expression. I talked frequently to him, even about the smallest things. To reduce his workload, I negotiated with the programme director to give him a week’s leave and mental health advice twice a year. I found that the training in our hospital was conducted effectively and the residents’ stress seemed to decrease thereafter as it acted as a bridge between them and their supervisors. (Case 10).*

#### 5. Providing support for prior personal problems

Sixteen out of the 39 residents (41%) had prior personal problems, such as mental health issues, insufficient social skills, and unprofessional behaviours. Coordinators provided support to these residents through various means.

##### 5–1. Fostering a better atmosphere for the mental health of residents

Residents struggled to adapt to the dynamic changes of roles and positions, especially during the transition from student life to resident life. The coordinators attempted to create a relaxed atmosphere for them and welcomed them to visit and interact. They took on the role of the main point of contact for residents with mental health issues, on behalf of busy supervisors, including listening to residents’ problems in a non-hierarchical equal relationship and keeping an eye out for them.

*Shortly after joining the programme he (*the resident*) fell ill and complained that he was not improving. I* (the coordinator) *assumed that he might have some mental health problem and tried to figure out the cause of his stress. Every morning, we took out time and talked to each other. His mental problem was due to concerns about a family problem as well as the training. I referred him to a specialist counsellor. I also shared his information with a small number of supervisors with his permission. He seemed to feel more comfortable talking to me. I thought we* (coordinators*) should be more careful during times of change in the resident’s environment, such as at the beginning of training. (Case 18).*

##### 5–2. Intervening for residents with insufficient social skills/unprofessional behaviour

Some residents suffered because of insufficient social skills or unprofessional behaviour, which were difficult to remediate. Many of the issues were related to multiple factors, including mental health problems, the training environment/programme, and partly societal norms. The coordinators provided remediation for these residents by arranging meetings with the medical team and communicating with affiliated organizations. They tried to maintain a non-hierarchical relationship, listened to the residents, and created a positive atmosphere, with a sense of good citizenship, but some of the cases were challenging to solve.

*The resident was asked by his supervisor to attend a conference and take a role of respondent, however, on the day of the conference, the resident suddenly cancelled and the supervisor had to ask another resident to take over the role. The resident insisted that he had not accepted the supervisor’s proposal and never apologised. As the atmosphere between them was bad, I* (the coordinator) *arranged an opportunity for them to talk to each other and share their feelings. The atmosphere of the meeting was mature and the relationship improved superficially, although I could not know the actual feelings between them. (Case 26).*

#### 6. Providing support for isolated residents

The distress of isolation paired with participation in an unfamiliar environment hindered residents’ socialization. Residents who graduated from a different medical school and were isolated from previous classmates had difficulties in adapting to the programme. The coordinators identified residents facing problems due to isolation from families, friends, and peers, supported them by inviting them to the office, creating a relaxed atmosphere, talking to them with a non-hierarchical attitude, and reassuring them.

*Her (*the resident’s*) mother was sick and died. After the funeral, she returned to work, but her grief could not be healed and she took an additional leave for three weeks. When she saw the patients brought in by ambulance, she remembered her mother and said it was hard for her. I* (the coordinator) *provided an environment where she could take a break, and her emergency duty was suspended for several months, after consulting with the director. As time passed, she regained her brightness, and a year after completing the training, she told me that she had got married. (Case 28).*

#### 7. Preventing problems with peers

The coordinators made efforts to prevent problems with peers or health professionals. They arranged for opportunities to foster communication between residents and peers, advised residents that peer evaluation was important, and facilitated the residents’ self-reflection.

*The resident seemed to be gentle, but I* (the coordinator) *noticed a problem with him when I received multi-source feedback from other health professionals. There were harsh comments such as “he is too loud” and “he is arrogant”. He denied these behaviours at the beginning. I tried to listen to his thoughts and reasons, and I explained the importance of evaluation by colleagues. We talked about it repeatedly. Afterwards, he was able to think about his colleagues and communicate well with the staff. The supervisors trusted him, and he became one of the best residents. I value the personality of each resident and try to be upfront with them so as not to hurt their pride. When the supervisors don’t want to say something or don’t want to get angry with the resident, I will go in the middle and convey the message from the supervisor to the resident. (Case 36).*

## Discussion

This is the first study investigating and detailing the educational roles of programme coordinators for the benefit of residents; specifically, the first one linking these roles to the critical aspects of residents’ PIF [17, 20]. We identified seven educational roles of coordinators for residents (Table 2). Our study suggests that coordinators perform valuable functions to identify the residents in difficulty and help with the development and PIF of residents. Programme coordinators have important roles beyond the scope of administration as suggested by Hu et al. [13], and these educational roles should be added to their role profiles or job description.

Among the socialization factors, clinical supervisors play central roles in trainees’ education, professional development, and PIF by facilitating clinical experiences, teaching formally and informally, and improving the training environment [17, 20]; however, it is sometimes difficult for the supervisors to handle all aspects of the socialization context that may influence residents’ professional development. Furthermore, clinical rotations during residency programmes are often short [9], and the relationship between supervisors and residents are sometimes hierarchical and not always positive. Programme coordinators are stable members of educational team who can observe residents during the entire training period in a non-hierarchical manner. Evidence suggests that coordinators’ behaviour is more collaborative, less competing, and less aggressive than of faculty physicians [26]. Coordinators can play an ‘ancillary’ but indispensable roles [9] that assess and foster residents’ PIF, help supervisors [2], and contribute to a better residency programme.

As for the frequency of factors and issues of residents supported by coordinators, prior underlying personal problems including mental health issues and insufficient social skills/unprofessional behaviours, were the most common, followed by isolation from family/friends/peers, problems with peers, and behaviours of supervisors/mentors (Supplementary Table). The National Survey of Internal Medicine Residency Program Directors in the US revealed that 42% of residents with difficulties had an underlying cause of situational, personal, or professional stress [27]. Reamy et al. reported that 9% of family medicine residents faced attitudinal problems, interpersonal conflicts, psychiatric illnesses, family stress, and relationship disruption [28]. Our study suggests that programme coordinators can support issues relating to personal identity and professional relationships. As Jennings and Slavin described the importance of community fostering ‘a culture of mutual appreciation’ for the wellness of residents with difficulties such as depression and burnout [29], and Daskivich et al. described the importance of engagement of all stakeholders within the learning community [30], programme coordinators would be essential members of these learning communities.

The seven key coordinator roles identified in this study (Table 2) are related to coordinators’ approaches and attributes; in particular, four attributes are overarching to these seven roles (Table 3).

**Table 3:** Suggested attributes for programme coordinators

1) Non-hierarchical relationships with residents	
2) Parenting attitudes to residents: supporting, fostering, and protecting	
3) Sensitive to early and subtle changes in residents	
4) Perspective of a citizen, as a member of the public	

First, a non-hierarchical relationship between coordinators with residents was often identified in our study (cases 18, 26, and 28), suggesting how this important attribute was overarching to their educational roles. Cruess et al. [20] described that the most influential factors in facilitating PIF are role models/mentors and the clinical/non-clinical experiences of individual residents; however, the supervisor-resident relationship is sometimes a hierarchical one, which may cause problems in the residents’ PIF. Our study delineated such cases. We previously reported that conflicts between biomedically-oriented supervisors and psychosocially-oriented residents could discourage residents’ PIF [10]. According to Hofstede, who identified and validated six dimensions of culture worldwide, Japan is a society with a relatively high-power distance [31], so a non-hierarchical relationship between clinical supervisors and residents may be more difficult to achieve than in the Western countries with lower power distance [32]. In such professional and cultural contexts, the coordinators can act as a bridge between supervisors and residents, and request supervisors to reconsider their teaching methods and their interactions with residents. However, when supervisors or residents themselves show a hierarchical attitude toward the coordinators due to professional biases [33], it becomes difficult to be effective.

Second, parenting attitudes, as described by Hu et al. [13], would also be an important attribute for coordinators. In our study, many residents with mental health issues, deficient social skills, and isolation from family and peers were reported, and coordinators presented parenting behaviours such as supporting, fostering, and protecting them (cases 5, 18, 28–33, and 36). Residents have to establish a new relationship with peers and supervisors once they graduate from medical schools and start working in different teaching hospitals. These relationships are sometimes not easily established, and residents may fear isolation and be sensitive to how they are perceived and treated by their new peers. The fear of isolation might be strong in Japan because of the cultural characteristics of collectivism, which emphasises familial or fellowship ties [31]. A qualitative study of paediatric supervisors in Japan revealed that ‘defending (protecting) residents’ was an important attribute of clinical teachers [34].

Third, sensitivity to early and subtle changes in resident behaviours could help to identify the need for early support and intervention. Many coordinators in this study noticed changes in residents’ appearance, not only when they went through mental health problems, but also a problematic relationship with supervisors, decreased self-confidence, and dissatisfaction with the programme (cases 3, 7, and 10). Supervisors are usually busy with their clinical responsibility, and the clinical rotations of residents are often short. Thus, staff may overlook such changes in appearance. Coordinators are able to observe residents over the entire period of training, so they can be sensitive to their facial expressions and mood, and can offer a place with a relaxed atmosphere for them to communicate openly and comfortably. Mental health problems are a global issue for health professionals [29, 30], and a correlation between long working hours and the incidence of depression has been reported [35]. Residents may not be aware of their own maladaptive responses to stress, and the surrounding staff is more likely to recognize resident stress [36]. In this sense, coordinators play an important role in detecting early signs of mental stress and fostering a more productive learning environment to mitigate and possibly prevent stressors to avoid deterioration of mental health.

Fourth, coordinators are able to observe and assess residents from the perspective of a good citizen and a member of the public. Although coordinators are now regarded as important members of the team training health professionals, they are able to assess resident behaviours more sensitively and efficiently than medical staff, through the lens of expectations of a member of the public and through societal norms. They are able to notice residents’ insufficient social skills or inadequate behaviours as professionals, as Nikels et al. reported [2], to drive communication and remediation (cases 21 to 27). Some of these behaviours could be learned as residents develop their skills and attitudes as a professional through the residency programme; however, some unprofessional behaviours are derived from prior personal experiences and awkward personal identities, which may be difficult to remediate.

This study suggests a wide range of educational roles for programme coordinators from a PIF standpoint and identifies some valuable exemplary attributes. Many of the proposed roles are not formally described or were under-acknowledged in the previous reports [2, 4, 5]. Flynn et al. conducted an action research on the support for trainees and clarified recommendations for administrative staff, including a communicative role, need for referral pathways for students in crises, peer support for coordinators, and skills training for mental health [37]. Based on these recommendations, they established a staff development programme for administrative and teaching staff using different scenarios, such as trainees with difficulties such as depression, absence without explanation, and unfamiliar distant clinical placement [37]; many of these scenarios are similar to those in our study. Thus, our study provide critical information that can lead to a revision of the roles and responsibilities of programme coordinators as well as the addition of supportive roles. Our study can also help to facilitate improvement of staff development programmes for coordinators and faculty.

## Limitations

We adopted a semi-structured and web-based anonymous questionnaire with the intent to collect as many cases of educational support by programme coordinators as possible not only from all over Japan but also from other countries for future studies, with consideration for confidentiality, sensitivity of the cases, and language barrier. Our sample may have been biased to those programs due to the low response rate. There could also be issues of selection as some cases were reported based on their sensitive nature, thereby impacting results. However, while select, this is an important sample to survey from the standpoint of identifying roles and attributes of the coordinators. Further studies should directly interview coordinators to confirm and deepen the findings of this study. Clinical supervisors’ and trainees’ perception of the roles of coordinators should also be investigated for triangulation of our results. Finally, we investigated resident cases in Japan, a country where cultural characteristics of collectivism and relatively high-power distance may affect the results, and limit its generalizability. Similar studies on the educational support provided by programme coordinators should be conducted in other cultural spheres.

## Conclusions

This study highlights the educational roles of programme coordinators in the PIF of residents. Coordinators play important educational roles by communicating with supervisors, protecting/facilitating/supporting residents, and fostering/creating a productive and safe learning environment. Attributes such as non-hierarchical relationships, parenting attitudes, sensitivity to changes in residents, and a citizen’s perspective underpin their educational roles. Our results provide evidence for revising role profiles of programme coordinators and providing targeted professional development training for coordinators.

## Supplementary Information

**Additional file 1: Supplementary table.** Supplementary table shows resident cases supported by the coordinators

## Data Availability

The datasets used and/or analysed for this study are available with the corresponding author upon reasonable request.

## Abbreviation

PIF: Professional identity formation
