# Supplementary material for: Do programme coordinators contribute to the professional development of residents? an exploratory study
Source: BMC Med Educ. 2022 May 18;22:381. doi: 10.1186/s12909-022-03447-y (PMC9118683; doi:10.1186/s12909-022-03447-y)
Supplement: Supplementary file 1 — Additional file 1: Supplementary table. Supplementary table shows resident cases supported by the coordinators [file 12909_2022_3447_MOESM1_ESM.docx]

**Supplementary Table**

No. Gender Socialization Issues of residents Coordinators’ responses

Factors*

1 M 1 Reprimands by the supervisor Listen to the resident, check the situation, report to the director

2 M 1 Strict supervisor Listen to the resident and the supervisor, report to the director

3 F 1 Strict supervisor, gloomy expression Provide mentoring, request supervisor to re-consider his teaching

4 M 1 Miscommunication, gloomy expression Create an approachable atmosphere

5 M 2 Medical error and a patient death Listen, watch, support by the hospital team, revision of teaching

6 M 3 Concerns about future practice Listen, watch, counselling, support family

7 F 3 Decreased confidence in procedural skills Reassurance, provide opportunities to practice

8 F 3 Decreased confidence of future roles Set of mentoring, create an approachable atmosphere

9 M 3 Decreased confidence of future practice Frequent interview, support family, re-scheduling

10 M 4 Overwork, dissatisfied expression Listen, counselling, improve the environment, bridge to supervisor

11 F 4 Inconsistent instructions Suggest review of instruction guide, support resident-centeredness

12 F 5-1 Psychological problem, self-injury Create an approachable atmosphere, reassure, re-scheduling

13 F 5-1 Poor mental health, unable to come to work Create an approachable atmosphere

14 F 5-1 Avoid and interruption of training Talk with the resident, bridge to supervisors, re-scheduling

15 M 5-1 Suspension of training, depression Talk with the resident, referral to psychiatry, re-scheduling

16 F 5-1 Frequent absence, gloomy expression Listen, talk, reassure, re-scheduling

17 M 5-1 Depression, absence after a patient’s death Listen, reassure, create an approachable atmosphere

18 M 5-1 Poor mental health, family concerns Create an approachable atmosphere, bridge to counsellor

19 M 5-1 Poor mental health, unable to work Referral to industrial physician, rehabilitation of working

20 M 5-1 Frequent interruption of training Interview, bridge to supervisors, re-scheduling

21 F 5-2 Passive student-like attitude Remediation education

22 M 5-2 Frequent late coming, poor attitudes Listen, talk, share information with supervisors

23 M 5-2 Poor motivation and communication Listen, watch

24 M 5-2 Absence without permission, loneliness Check physical condition, counselling, re-scheduling

25 M 5-2 Absence without permission, arrogance Remediation education

26 M 5-2 Sudden cancellation of promises Listen, remediation, conversation with supervisors

27 F 5-2 Frequent absence, disregard appointments Create an approachable atmosphere, counselling

28 F 6 Mother’s death, flashbacks Listen, re-scheduling

29 F 6 Childbirth, interruption, gloomy expressions Listen, reassure, career advice

30 F 6 Graduate of different school, gloomy expressions Reassure, create an approachable atmosphere

31 F 6 No peers, gloomy expressions Creating an approachable atmosphere, cuddle with her

32 F 6 Isolation from peers due to childcare Re-scheduling, share with medical staff

33 M 6 Isolation from peers and supervisors Facilitate communication with peers, experienced mentors

34 M 7 Maltreatment by peers due to mid-carrier Facilitate communication with peers, the opportunity for interaction

35 F 7 Mix up official affairs with personal ones Advice to make her aware of the peers' evaluations

36 M 7 Negative evaluation by peers, arrogant attitude Advice to make him aware of the peers' evaluations

37 F 7 Trouble with medical staff and patients Confirmation of situation, report to the supervisor

38 M 7 Harassing communication from senior staff Listen, facilitate self-reflection

39 F 7 Opposition from peers due to easiness on herself Facilitate self-reflection

M: male; F: female

* Socialization factors: 1. Supervisors/mentors; 2. Clinical/non-clinical experiences; 3. Formal teaching/self-assessment; 4. Learning environment; 5. Prior personal problems (5-1. Mental issues; 5-2. insufficient social skills/unprofessional behaviour); 6. Isolation from family/friends/peers; 7. Problems with peers
